# Integrating multiple information sources for landslide hazard assessment: the case of Italy

**DOI:** 10.1038/s41598-022-23577-z

**Published:** 2022-12-01

**Authors:** Rachele Franceschini, Ascanio Rosi, Matteo del Soldato, Filippo Catani, Nicola Casagli

**Affiliations:** 1 Department of Earth Sciences, University of Florence, Via Giorgio La Pira, 4, 50121 Florence, Italy; 2 Department of Geosciences, University of Padova, Via G. Gradenigo, 6, 35131 Padua, Italy; 3 National Institute of Oceanography and Applied Geophysics-OGS, Borgo Grotta Gigante n. 42/c, Sgonico, Trieste Italy

**Keywords:** Natural hazards, Geomorphology, Statistics

## Abstract

Landslides are the most frequent and diffuse natural hazards in Italy causing the greatest number of fatalities and damage to urban areas. The integration of natural hazard information and social media data could improve warning systems to enhance the awareness of disaster managers and citizens about emergency events. The news about landslide events in newspapers or crowdsourcing platforms allows fast observation, surveying and classification. Currently, few studies have been produced on the combination of social media data and traditional sensors. This gap indicates that it is unclear how their integration can effectively provide emergency managers with appropriate knowledge. In this work, rainfall, human lives, and earmarked fund data sources were correlated to “landslide news”. Analysis was applied to obtain information about temporal (2010–2019) and spatial (regional and warning hydrological zone scale) distribution. The temporal distribution of the data shows a continuous increase from 2015 until 2019 for both landslide and rainfall events. The number of people involved and the amount of earmarked funds do not exhibit any clear trend. The spatial distribution displays good correlation between “landslide news”, traditional sensors (e.g., pluviometers) and possible effects in term of fatalities. In addition, the cost of soil protection, in monetary terms, indicates the effects of events.

## Introduction

The vulnerability of the population and the effects of natural hazards depend on the level of socioeconomic development but also on the geological and geomorphological settings of an area. A recent report from the US National Academy of Science recognized a growing threat in relatively wealthy municipalities in the United States, with a higher risk to populations with less protection from insurance or the social safety net^1^. Production growth and accelerated urbanization, as well as the concentration of the population and enterprises in hazardous zones, are the main factors of the risk increase^2–4^. Climate change is expected to make these conditions even worse^5,6^. In fact, extreme weather events are predicted to increase in frequency and severity at regional and local scales^7^. Kiely^8^ affirmed that rainfall with a heavy intensity rate is increasing, with a return period that is shortening from 30 to 10 years. Consequently, the frequency and severity of natural disasters on the economy are growing. The economic impact and human losses outlined as a consequence of natural disasters are necessary tools to evaluate and develop measures for risk reduction. Several research projects have examined the economic impact of specific disaster events^9^. The economic damage from natural disasters is higher the wealthier the affected country^10^. The possible involvement of urbanized areas by landslides is an issue faced by governments and private actors. These authorities can adopt measures to prevent, or at least mitigate, economic losses. All other things being equal, countries of larger economic size will have more wealth potentially destroyed and are therefore expected to experience larger losses. Disaster prevention and mitigation measures are costly. Both private actors and governments can more easily finance them in richer countries rather than in low social capital countries^10^. Hurricane Katrina was the costliest natural disaster ever with an estimated economic loss between 82 billion^11^ and 150 billion US$^12^, and 986 victims^13^. In Italy, during October 2014, one flash flood caused several landslides and mudflows in Genova, causing 1 dead, 300 million euros of damages and 250 people homeless^14,15^.

Landslides in Italy are the most frequent and diffuse natural hazards causing the greatest number of fatalities and damage to urban areas^16,17^. Landslide research chiefly relies on landslide inventories for a multitude of spatial, temporal or process analyses^18–20^. The forecasting and monitoring of landslides are being increasingly characterized as a problem of “big data” since there are different data sources that can be used to support decision-making, such as satellites^21–27^, rainfall gauges^28,29^ and hydrological networks^30^. The retrieval of data, using specific data mining algorithms, from technical reports and/or newspapers can further extend the exploitable data. The use of social media in detecting natural hazards has shown promising results^31,32^. The joint analysis of data from different social media can help to capture disaster situations with a relatively high temporal and spatial resolution to map different events, such as landslides, across various locations^33–35^. Most empirical literature that employs social media data has focused on investigating the relationships between social media data and real-world phenomena, which involves extracting aggregated, thematic and spatiotemporal patterns from social media activity. These data have often been used as proxies for a variable of interest and correlated with conventional data sources, such as physical sensors and survey data^36^. Currently, few studies on the combination of social media and other data sources have been produced. It thus remains unclear how social media data can (i) be effectively integrated with hazard-monitoring data and (ii) provide emergency managers with appropriate information for better land-use planning and early warning support^37^.

Social media information about floods and forest fires has been combined^38,39^ with rainfall data, satellite imagery, and wind velocity. Such studies have demonstrated the efficiency of using social media as an additional source of information usable for near-real time forecasting and policy decision-making. Franceschini et al.^40^ used a set of newspaper articles to obtain information about the spatial and temporal distribution of landslide events in Italy between 2010 and 2019. The news was collected by the semantic engine to classify and geotag news (SECaGN) algorithm developed by Battistini et al.^41,42^. The algorithm searches and catalogues information about recent landslides in online newspapers. This dataset was used to obtain correlations between landslide events, rainfall data and direct consequences in terms of socioeconomic losses (human lives and money) for the whole Italian territory.

The goal of this manuscript is to show how social media, in combination with other data sources, can be useful to better define the landslide hazard of a territory and for assisting authorities and helping the enhancement of the landslide early warning system (EWS) at the national scale. Evaluations can be important to understand and measure the impact of natural disasters, as well as to plan the best strategies for risk reduction.

## Study area

Italy is a European country exposed on the Mediterranean Sea and covers an area of approximately 300,000 km^2^. Italy is divided into 20 regions and is then subdivided into 107 provinces and 7926 municipalities. It is also divided into 158 warning hydrological zones (WHZs), which represent the reference territorial unit for natural hazard-warning purposes. The Italian territory is characterized by the Alpine and Apennine chains in northern and central and southern of Italy, respectively. Italy is the European country with the widest areal distribution and highest recurrence of large landslides, causing severe losses of lives and goods^43–45^. Currently, the IFFI database (Italian Inventory of Landslide^46^) includes over 600,000 landslides, affecting an area of 23,700 km^2^, representing 7.9% of the national territory^47^. Every year, thousands of landslides occur in the national territory, and a few hundreds of these result in victims, casualties, evacuations and damage to buildings, cultural heritage, and primary transportation infrastructure. For example, in 2017, 172 events were reported and 146 events occurred in the previous year^47^. Legambiente^48^ surveyed 1181 extreme weather events from 2010 to the present that caused damage in Italy. A total of 637 municipalities (8% of the total) have recorded events with relevant impacts. In terms of human lives and injuries, 264 people have been victims of natural disasters. The CNR (National Research Council) has recorded the evacuation of over 27,000 people due to events such as landslides and floods between 2016 and 2020, which becomes 320,000 people counting the events that have occurred since 1971. The regions most affected by extreme events since 2010 are Sicily and Lombardy, with 144 and 124 events, respectively^40^.

## Materials and methods

**Figure 1:**
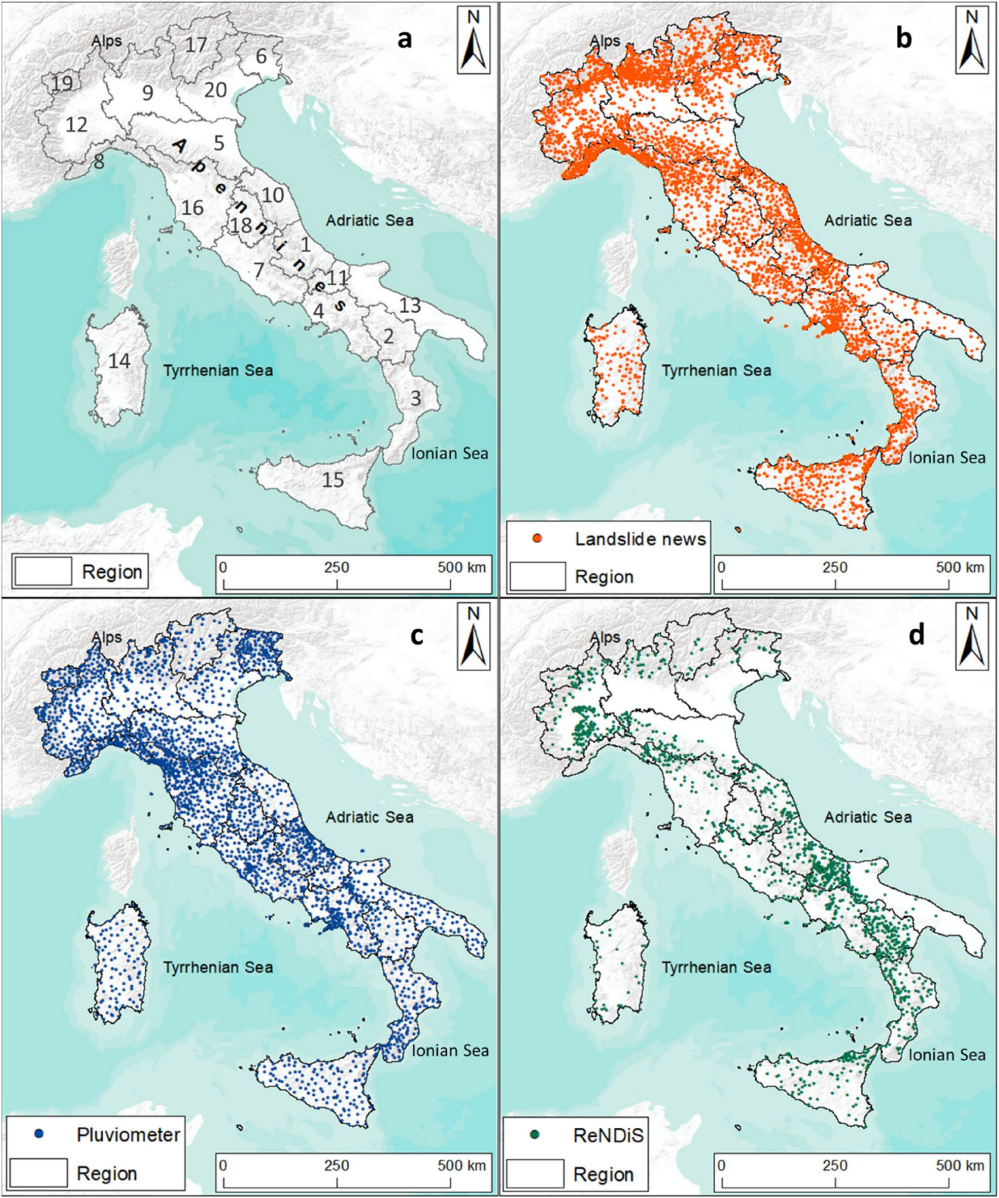
(**a**) Italian regions, 1: Abruzzo, 2: Basilicata, 3: Calabria, 4: Campania, 5: Emilia Romagna, 6: Friuli Venezia Giulia, 7: Lazio, 8: Liguria, 9: Lombardy, 10: Marche, 11: Molise, 12: Piedmont, 13: Puglia, 14: Sardinia, 15: Sicily, 16: Tuscany, 17: Trentino Alto Adige, 18: Umbria, 19: Valle d’Aosta, 20: Veneto; (**b**) Distribution of “Landslide news”; (**c**) Rain gauges across the Italian territory; (**d**) ReNDiS data for landslide events. The map was generated using ESRI ArcMap 10.8.1 (https://www.arcgis.com/home/item.html?id=33064a20de0c48d2bb61efa8faca93a8).

Four datasets representing different sources of information were analysed in this work: (i) online newspaper, (ii) rainfall data, (iii) population at risk from landslides and floods in Italy (Polaris) and (iv) the National Repository of Soil Defense interventions (ReNDiS) inventory. Newspapers can be used to create a landslide inventory, which, in turn, can be analysed for landslide hazard assessment (e.g., landslide distribution, frequency and intensity^40^). Polaris and ReNDiS identify the event effects in term of human lives, involved regions and earmarked funds for remediation and risk mitigation work (Fig. 1).

The analysed temporal interval covered 10 years (2010–2019) and the whole Italian territory was considered. Regions (Fig. 1a) and warning hydrological zones (WHZs) were the reference units used to carry out the analyses.

### Landslide news

The landslide news dataset was gathered from Franceschini et al.^40^, in which 174,616 “newspaper articles” referring to 31,878 “landslide news” were collected and analysed (a landslide can be reported by several articles). The newspaper articles were automatically mined by the SECaGN algorithm^41,42^ from 2010 to 2019 over the whole Italian territory (Fig. 1b). The data mining occurred within Google News, as it considers with more completeness national and local newspapers^40^.

### Rainfall data

A dense network of spatially distributed rain gauges over Italy provides continuous direct observations of rain measurements for several specific locations^49^. This network consists of over 4500 rain stations, which provide rainfall measurements approximatively every 15 min (Fig. 1c).

**Figure 2:**
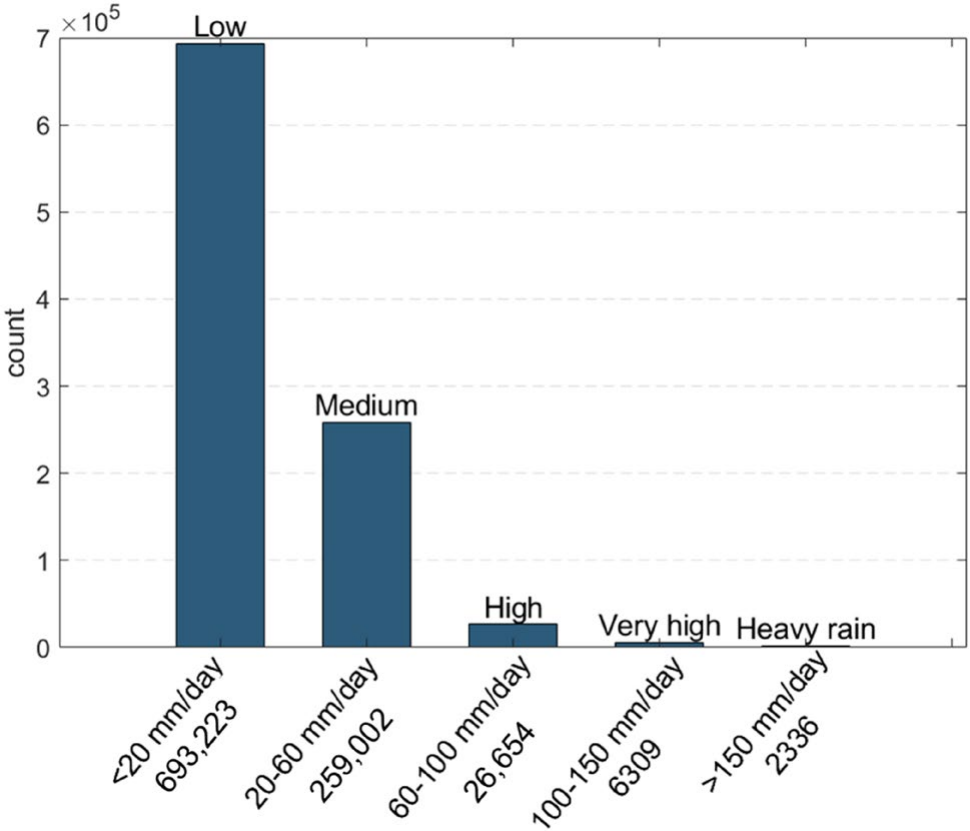
Rainfall data between 2010 and2019 (from Del Soldato et al.^50^). For each class, the number of occurrences was calculated (event count).

This network was deeply analysed by Del Soldato et al.^50^. Data were analysed to select only the rain gauges recording data for more than 20 h per day (to remove low representative data) and to remove noisy data (e.g., negative rainfall values or values higher than 400 mm/h). The database appears to be statistically robust. The analysis was carried out from data covering the 2010–2019 period. The same authors divided the rainfall events into five classes (according to the classification used by the Italian Civil Protection Department) based on their average intensity (as mm/day). In addition, for each class, the number of events over the analysed period was calculated (Fig. 2).

### Population at risk from landslides and floods in Italy—Popolazione a Rischio da Frana e da Inondazione in Italia—Polaris

Polaris is a website managed by the Research Institute for Hydrogeological Protection (IRPI) of the National Research Council (CNR) of Perugia (Italy). The main aim is to attempt to assess the geo-hydrological risk to the Italian population. For years, the IRPI has collected and processed historical information on landslides and floods that have caused damage to the population. Every year, Polaris produces a report with various statistics concerning the distribution of injuries, deaths and evacuated and missing people due to these natural events. For landslide events the database provides spatial information about the involvement of municipalities, provinces and regions. The dataset has information since 2011, which was the year that the project started; hence, no data were collected in 2010.

### The National Repository of Soil Defence interventions—ReNDiS

ReNDiS is a database of remediation projects planned to restore the damage caused by natural events such as landslides and floods and was founded by the Italian government^17^. Data are collected through continuous contacts between ISPRA (*Istituto Superiore per la Protezione e la Ricerca Ambientale*—Italian Institute for Environmental Protection and Research) technicians and local authorities managing the projects in the whole Italian territory. Therefore, through the ReNDiS database, the Italian government can be informed in real time of how funds for risk mitigation projects are being spent and how they are distributed across the country^17^. The ReNDiS dataset started in 2000 and covers the period until 2020. Overall, more than 2.7 billion euros were allocated as funding to remediate the structural damage and economic losses. For the analysis conducted in this work, the interval of intervention funding from 2010 to 2019, the involved region, the landslide events, and the incurred expenses were considered (Fig. 1d).

### Methods

**Figure 3:**
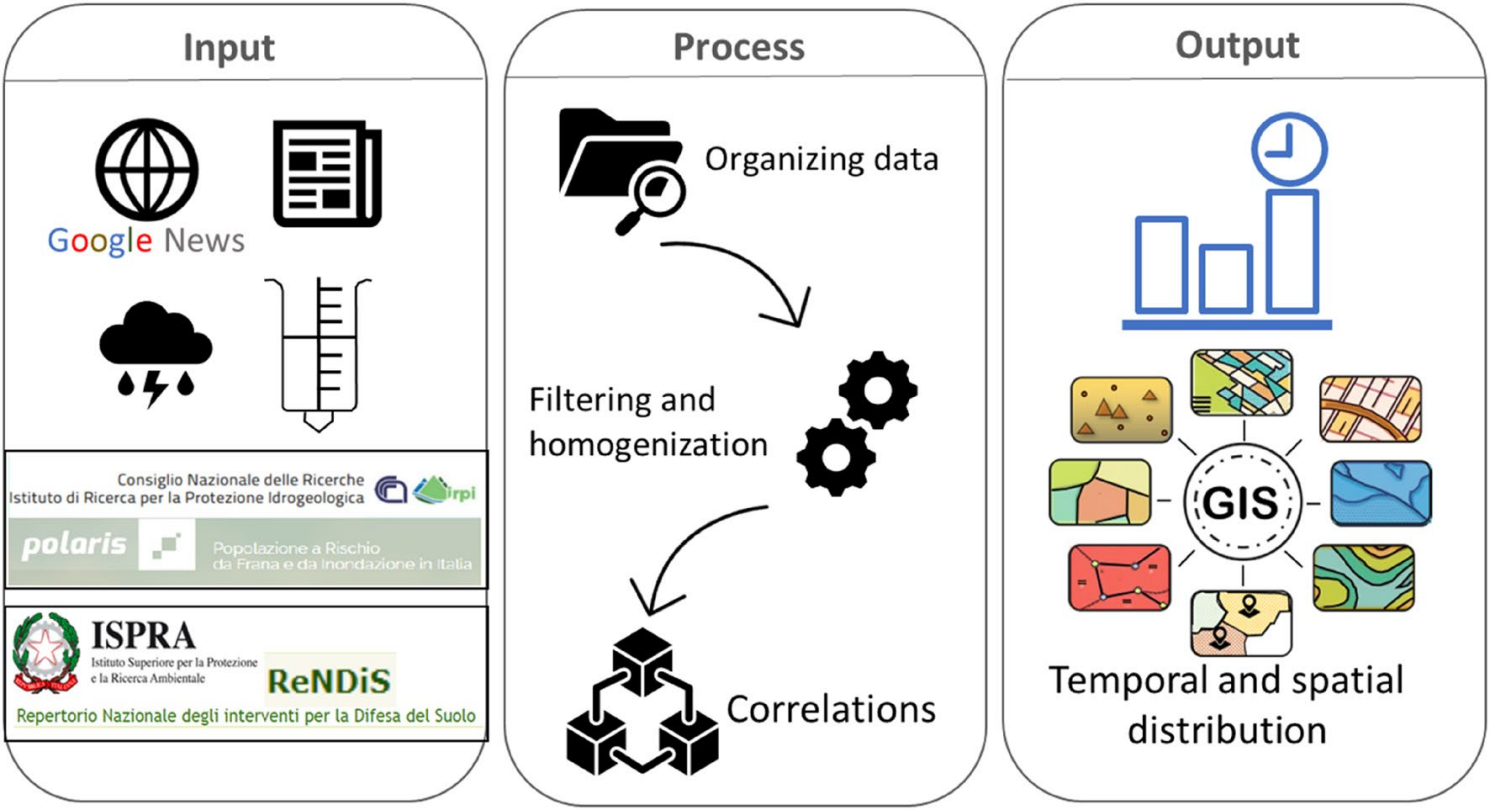
Workflow of the work. The output corresponds to panels and maps to obtain information on approximately 10 years of landslides for the whole Italian territory.

The four datasets were analysed and compared. The aim was to outline the distribution and evolution of landslide hazards and their effects in the Italian territory (Fig. 3). All datasets were analysed, filtered and homogenized to obtain correlations with each other. The outputs consist of panels and maps, which describe the temporal and spatial distribution of landslide events over Italy in 10 years.

Each dataset provides specific kinds of data, which are organized as described below:

- “Landslide news”—data from social media were used as proxies to correlate rainfall data and other available datasets. The social media database contains two pieces of information: (i) “newspaper articles” and (ii) “landslide news”. “Newspaper articles” are the number of articles published for each landslide event from different newspapers. This information outlines the media impact of the event; the higher the number of publications is, the higher the intensity and the impact of the event. “Landslide news” refers to articles considering the same landslide event, which are grouped in a single datum (i.e., several newspaper articles referring to the same landslide event are considered as 1 landslide news event). This information can delineate the hazard in a certain area. A higher number of 'landslide reports' indicates a greater propensity for landslides in an area.
- Rainfall data—they were classified by Del Soldato et al.^50^ into five classes based on daily intensity. Among these classes, “high intensity” (60–100 mm/day), “very high intensity” (100–150 mm/day) and “heavy rain” (> 150 mm/day) show a clearer spatial distribution with respect to “medium intensity” (20–60 mm/day) and “low intensity” (5–20 mm/day). For this reason, the frequencies of the rainfall events of these classes were considered (hereafter named “relevant rainfalls” hereafter). Two databases were gotten: the frequency of each intensity class and the count of rainfall events.
- Polaris—starting from annual Polaris reports, two different datasets were created: (i) The Injured, Deaths, Evacuated and Missing people (IDEMs) dataset was used for the temporal distribution analysis of human involvement and (ii) the Injured, Deaths and Missing people (IDMs) dataset was used for the spatial distribution investigation. This distinction was made because of the lack of spatial information about evacuated people.

The first step of the analysis aimed to outline the temporal evolution of each dataset from 2010 to 2019.

In the second step, each dataset was analysed to obtain the spatial distribution at the regional and WHZ (Wheatear Hazard Zone) scales. Regional scale analysis aimed to have an overview of the spatial distribution of the considered variables: (i)” landslide news”, (ii) “newspaper articles”, (iii) rainfall intensity, (iv) rainfall events, (v) IDMs, and (vi) funds. Then, the percentage of hazardous landslide areas and the percentage of buildings at risk^51^ of each region were correlated to earmarked funds for soil protection. The percentages were scaled on the basis of the regional size with respect to the Italian territory (300,000 km^2^) and to the building number of the whole national territory (12,187,698 buildings according to the National Institute of Statistics).

At the WHZ scale, a more detailed analysis of landslide news and rainfall events was made. Regarding rainfall data, the frequency of the 3 intensity classes, “high”, “very high” and “heavy rain” and the number of events in each class (event count), were correlated.

## Results

Several organizations create reports or datasets describing many different aspects of natural disasters. In this work, four 10-year-long datasets (2010–2019) on landslide events in Italy were analysed. The analysis was carried out to obtain information and to determine the spatial, at regional and WHZ scales and the temporal correlations of the available data. Overall, the analysed data were thus divided into 31,878 “landslide news” with 174,616 “newspaper articles”, 2040 rain gauges with 35,299 rainfall events, 198 data from Polaris and 1539 data from ReNDiS. All datasets cover the 2010–2019 period, except the Polaris dataset, since it started from 2011.

### Temporal distribution

From a temporal point of view, each Italian region experienced some landslides in the investigated period, with approximately 1477 IDEMs per year.

“Landslide news” showed an increasing trend from 2010 to 2014, which was repeated in the 2015–2019 period.

**Figure 4:**
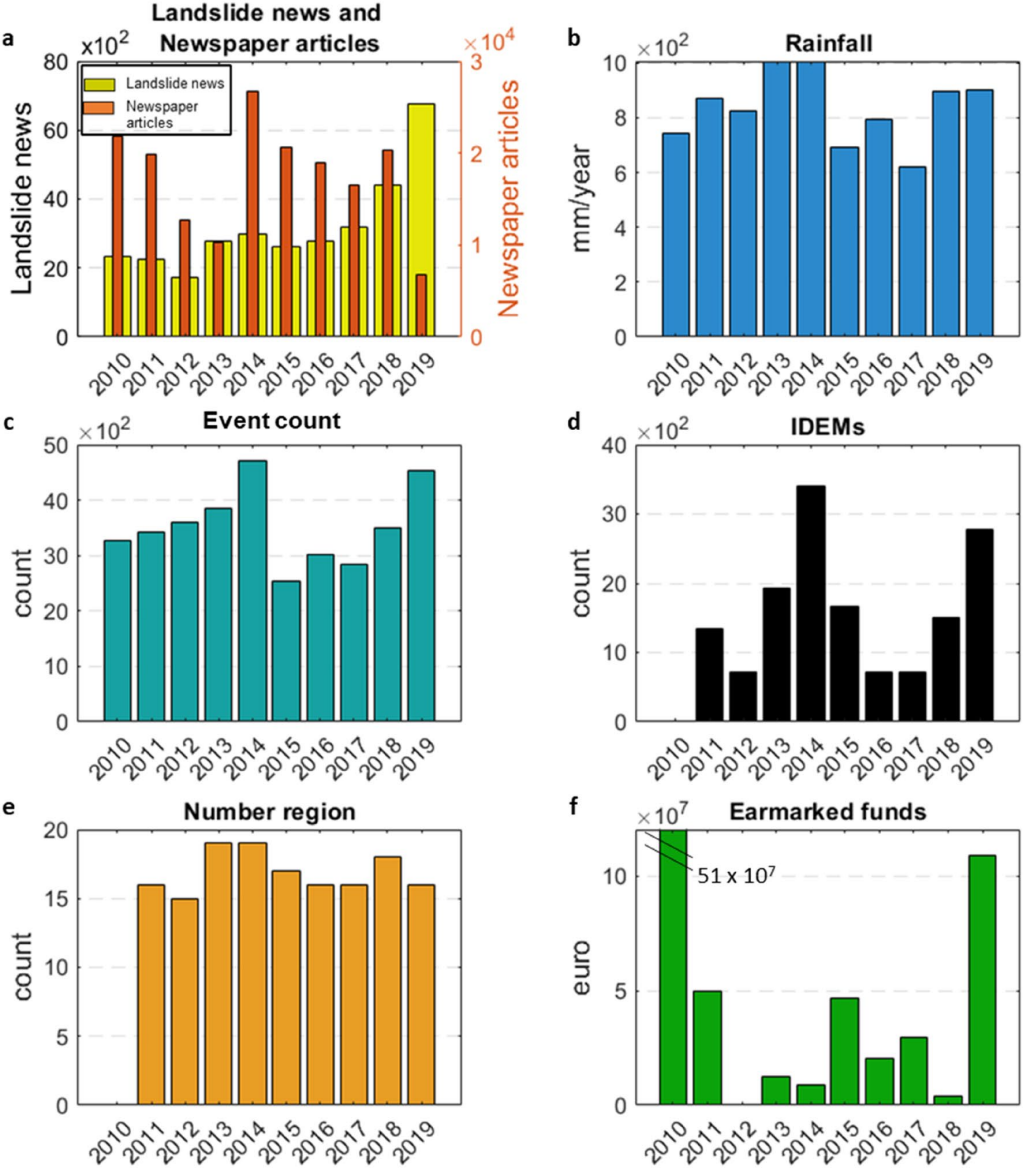
Temporal distribution with different information: (**a**) “Landslide news” and “newspaper articles” from social media. (**b**) Rainfall data with mm/y and in (**c**) event count. (**d**) Polaris with IDEMs (injured, death, evacuated and missing) number. (**e**) Involved region. (**f**) Earmarked funds for soil protection (euro—ReNDiS with focus for better vision).

At the end of the first quinquennium, 2014 featured by the highest number of “landslide news” (2988), with a mean rainfall of 1007.6 mm/year (Fig. 4a,b). 2014, with 4706 rain events, was also the year with the highest number of relevant rainfall events (Fig. 4c). In this year, 19 regions out of 20 were involved in landslides with 3406 IDEMs (Fig. 4d,e) as consequences. In contrast, 2014 was the second year with less earmarked funds for soil protection, with almost 8 million euros, second only to 2012, with just 227 thousand euros (Fig. 4f).

In the second quinquennium, the year with the highest number of “landslide news”, with 6775 data, was 2019. Although the rainfall data were constant with respect to the previous year, 2019 was the second year with a higher event count, with 4529 relevant rain events (Fig. 4a–c). In the same year, 16 regions were affected by landslides with 2775 IDEMs (Fig. 4d,e). 2019, in these five years, showed the highest values of earmarked funds, almost 109 million euros (Fig. 4f).

In general, “landslide news” increased from 12,083 news items in the 2010–2014 period to 19,795 in 2015–2019, while for rainfall events, the trend was inversed. The relevant rainfall event count decreased from 18,858 for 2010–2014 to 16,441 for 2015–2019. The event count passed from 3771 events/year to 3288 events/year. A similar trend was noticed for the rainfall data, which decreased from 4451 mm/5 years to 3910 mm/5 years. The same decrease was observed in “newspaper articles”, IDEMs and reported expenses between the 2010–2014 and 2015–2019 periods. “Newspaper articles” were 91,439 for the first quinquennium and 83,177 in the second, while the IDEMs distribution showed small decrease from 7402 to 7372.

### Spatial distribution

The “landslide News” can identify the municipality of a landslide event but not the exact location of the event. For this reason, the data were grouped on regional and WHZ bases to outline the areas with a higher number of published articles. The number of ”landslide news” was used as a proxy to identify those areas more affected by landslides, hence the most hazardous areas, in the observed period. The number of “newspaper articles” was used as an estimator of the intensity of landslides; the greater the effect of the landslide was, the greater the media echo.

**Figure 5:**
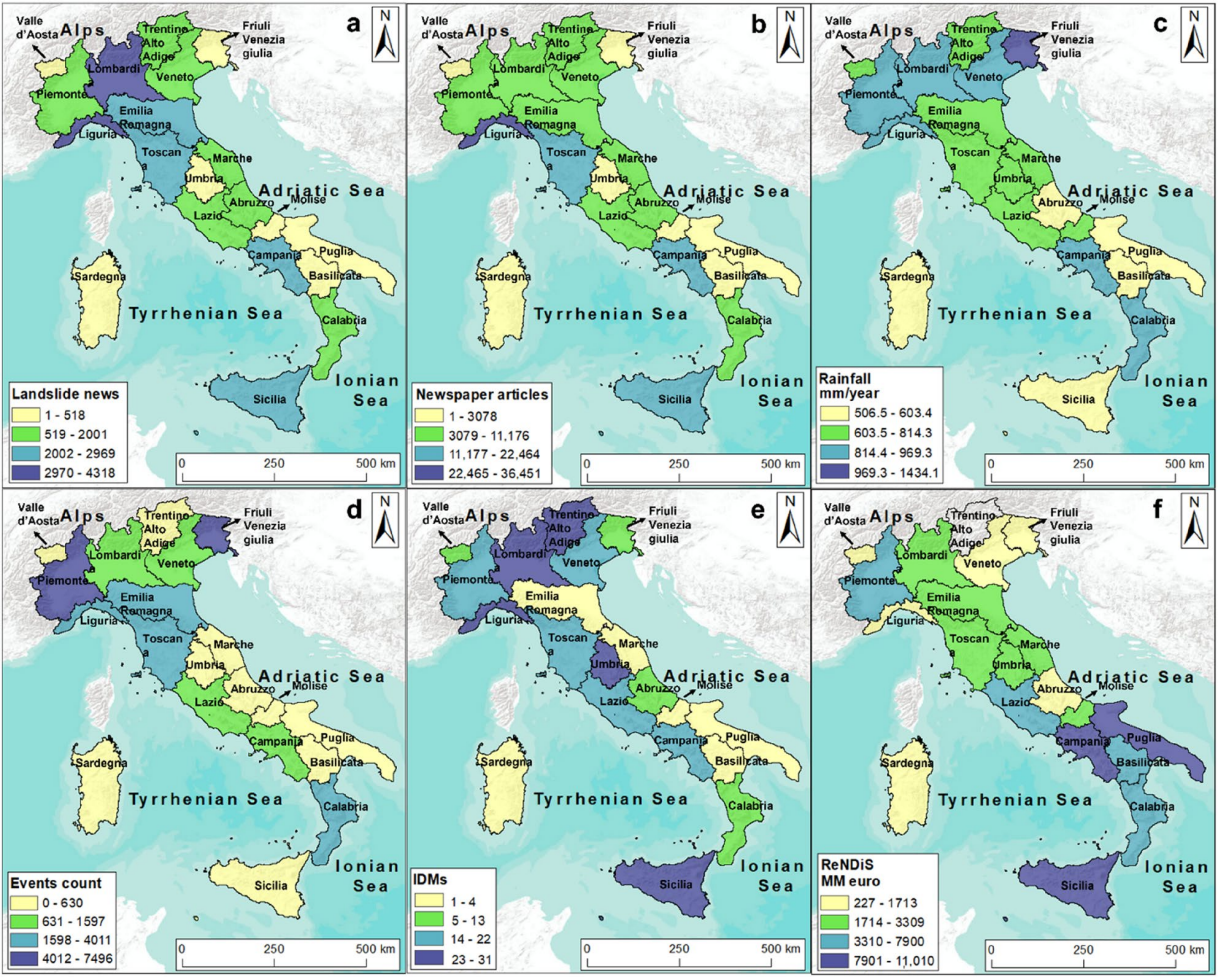
(**a**) Regional aggregation with “landslide news”, estimating landslide hazard; (**b**) regional aggregation with media impact, estimating landslide intensity; (**c**) regional distribution with rainfall data for 10 years (mm/10 years); (**d**) regional aggregation of relevant rainfall event counts (events display relevant differences in spatial distribution); (**e**) regional aggregation with IDMs from the Polaris dataset (the period covers only 9 years, from 2011 to 2019); (**f**) regional aggregation with earmarked funds for soil protection from ReNDiS (euro/10 years) for 10 years, considering landslide events. The map was generated using ESRI ArcMap 10.8.1 (https://www.arcgis.com/home/item.html?id=33064a20de0c48d2bb61efa8faca93a8).

The regions most affected by landslide events were mainly in the northern portion of the country. Liguria and Lombardia were the regions with the highest number of “landslide news” (Fig. 5a). A similar spatial distribution was achieved considering “newspaper articles” (Fig. 5b), rainfall data (Fig. 5c), relevant rainfalls distribution (Fig. 5d) and IDMs (Fig. 5e). Indeed, northern regions recorded the highest mean annual rainfall and number of relevant rainfalls with respect to central or southern regions, except for Campania and Calabria, where rainfall was similar to that in the northern part of the country. Friuli Venezia Giulia and Piedmont were the rainiest region of Italy.

IDM values were more evenly distributed across the country, but a higher number of IDMs was reported in the northern area of the country. For example, Trentino Alto Adige, Lombardy and Liguria were the three regions with the highest IDM numbers, 31, 30 and 27 respectively. No region showed 0 IDM after a landslide. Basilicata, Molise and Puglia showed only one IDM in agreement with low values of landslide events and rainfall data.

Figure 5f, which represents the earmarked funds for each region, shows that the Campania, Sicily and Puglia regions stand out for the highest fund allocation in the country, followed by Basilicata and Calabria.

In general, the distribution of “landslide news” and “newspaper articles” agreed with the number of relevant rainfall events and IDMs. In some cases, it was in contrast with earmarked funds. In fact, the earmarked funds for soil protection outlined more investments in southern Italy than in northern Italy.

Valle d’Aosta, Molise and Abruzzo are the Regions that exhibited the highest coherence between the analysed variables. In this sense, the morphology of the territory, the climatic setting, the size of the region and the density of people and buildings at risk can bias the distribution of landslide events.

In conclusion, Valle d’Aosta, Piedmont, Liguria, Lombardy, Veneto, Emilia-Romagna, Tuscany, Marche, Abruzzo, Lazio, Molise, Campania, Sardinia are the regions that had a higher correlation between the datasets. Conversely, Friuli-Venezia Giulia, Trentino Alto Adige, Umbria, Puglia, Basilicata, Calabria and Sicily were the regions with a lower correlation.

**Figure 6:**
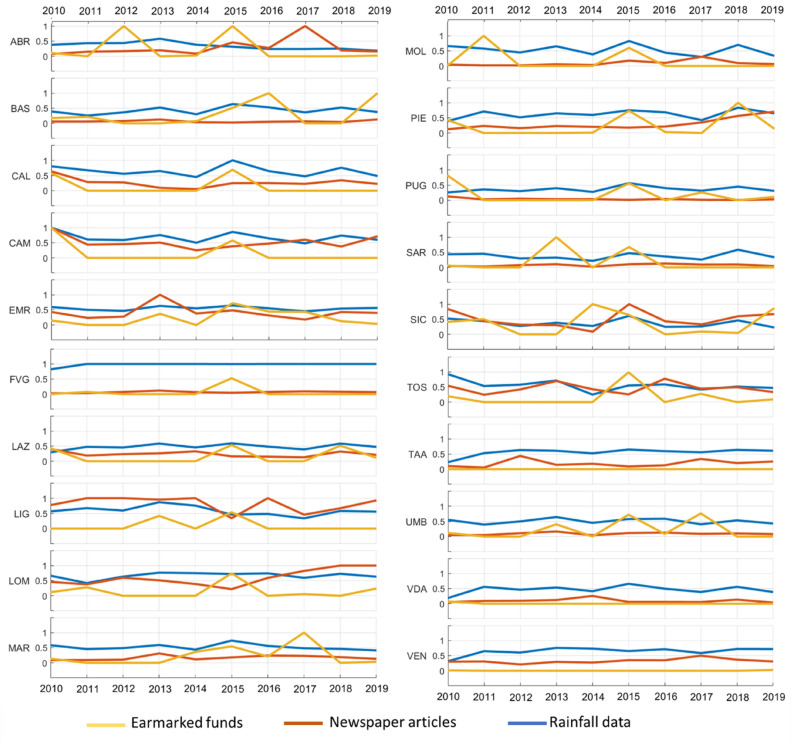
“Newspaper articles”, rainfall distribution and earmarked funds were normalized and correlated for each region for 10 years. *ABR* Abruzzo, *BAS* Basilicata, *CAL* Calabria, *CAM* Campania, *EMR* Emilia-Romagna, *FVG* Friuli-Venezia Giulia, *LAZ* Lazio, *LIG* Liguria, *LOM* Lombardy, *MAR* Marche, *MOL* Molise, *PIE* Piedmont, *PUG* Puglia, *SAR* Sardinia, *SIC* Sicily, *TOS* Tuscany, *TAA* Trentino-Alto Adige, *UMB* Umbria, *VDA* Valle d’Aosta, and *VEN* Veneto.

“Newspaper articles”, rainfall distribution and earmarked funds were normalized on the basis of their annual maxima to analyse and compare the variation of their values over time for each region. Figure 6 shows the trend of each variable for each region over 10 years. Regarding the northern regions, Trentino Alto Adige, Valle d’Aosta and Veneto did not show variations in earmarked fund distribution. Friuli Venezia Giulia, Lombardy and Piedmont revealed the same distribution of earmarked funds during 2015 (Fig. 6FVG, LOM and PIE). The Piedmont region presented a sharp increase in investment for soil protection in 2018. The Lombardy, Piedmont, Trentino Alto Adige, Valle d’Aosta and Veneto regions showed the same trend between “newspaper articles” and rainfall distribution: lower rainfall values corresponded to a lower media impact of landslide events (Fig. 6LOM, TAA, VDA and VEN). In contrast, for Friuli Venezia Giulia, it was possible to recognize high values of rainfall but very low values of media impact of landslide events (Fig. 6FVG).

Abruzzo, Emilia Romagna, Lazio, Marche, Sardinia, Tuscany and Umbria were the regions with a good correlation with the annual distribution of the variables. In fact, the “newspaper articles” number increased or decreased with rainfall data, and earmarked funds increased in the same year or in the following years of the landslide events (e.g., Abruzzo in 2012 and 2015 (Fig. 6ABR); Emilia Romagna in 2010, 2013, 2015, 2016 and 2017 (Fig. 6EMR); Lazio in 2010, 2014, 2015 and 2018 (Fig. 6LAZ); Marche in 2013, 2014, 2015 and 2017 (Fig. 6MAR); Sardinia in 2013 and 2015 (Fig. 6SAR); Tuscany in 2010, 2013, 2015 and 2016 (Fig. 6TOS); and Umbria in 2013, 2015 and 2017 (Fig. 6UMB)).

Basilicata, Calabria, Campania, Molise, Puglia and Sicily showed a good correlation between variables for 10 years. Basilicata and Puglia evinced similar trends for each variable. All of them revealed low values of “newspaper articles” but a good correlation between rainfall data and earmarked funds. In fact, high values of rainfall were measured during 2013, 2015 and 2018, with an increase in earmarked funds in the same year (e.g. Puglia in 2015, Fig. 6PUG) or in the next year (Basilicata in 2016 and 2019 and Puglia in 2017 in Fig. 6BAS and PUG). Calabria, Campania, Molise and Sicily presented similar distributions for each variable. For example, during 2010, “newspaper articles”, rainfall data and earmarked funds revealed high values in each region except for the Molise. During 2013, the increase in newspaper articles coincided with the increase in rainfall data but corresponded to low values of earmarked funds (fund increases started from 2014 in Sicily and only in 2015 in the other regions). 2015 and 2018 were the most coherent; in fact, all southern regions featured high values of rainfall data and “newspaper articles”.

### Correlation with hazard maps

**Figure 7:**
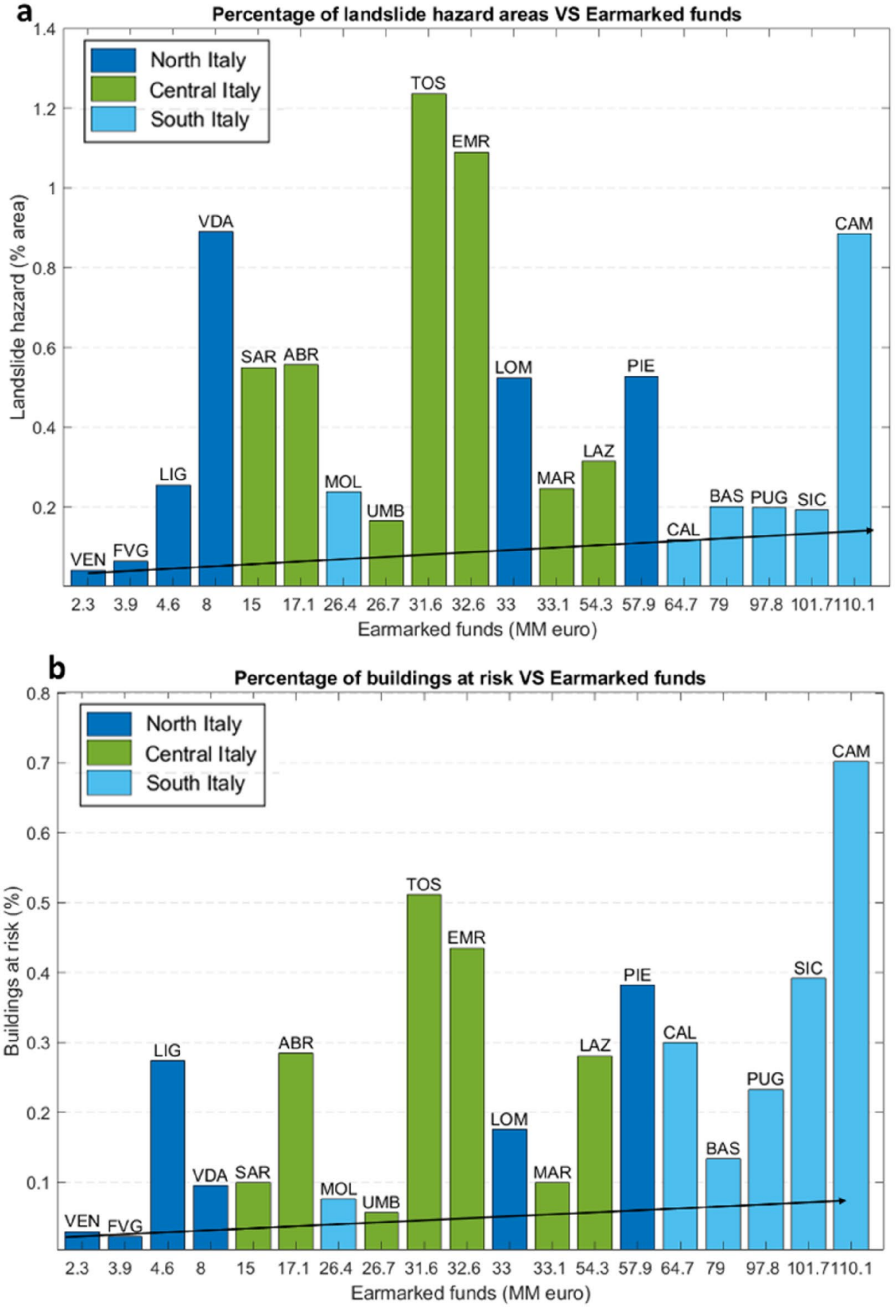
Validation data between earmarked funds in (**a**) with the percentage of landslide hazard areas for each region; in (**b**), the percentage of buildings at risk for each region. Each percentage was provided by ISPRA and normalized on the basis of regional size. *ABR* Abruzzo, *BAS* Basilicata, *CAL* Calabria, *CAM* Campania, *EMR* Emilia-Romagna, *FVG* Friuli-Venezia Giulia, *LAZ* Lazio, *LIG* Liguria, *LOM* Lombardy, *MAR* Marche, *MOL* Molise, *PIE* Piedmont, *PUG* Puglia, *SAR* Sardinia, *SIC* Sicily, *TOS* Tuscany, *TAA* Trentino-Alto Adige, *UMB* Umbria, *VDA* Valle d’Aosta, and *VEN* Veneto. The arrow indicates the increasing direction of allocated funds.

After the evaluation of the spatial and temporal distribution of the data, the correlation between the earmarked funds and the percentages of hazardous areas and buildings at risk in each region was analysed (Fig. 7).

Since the extent of the Italian regions varies greatly, the percentages of hazardous areas and buildings at risk were scaled with respect to the area of the Italian territory and to the total number of buildings.

The analysis was carried out considering the distribution of funds at the regional scale. By correlating these three variables, it was possible to outline which regions showed a higher percentage of both territory subject to landslide hazard and buildings at risk compared with the funds allocated for soil protection. For these analyses, the Italian regions were split according to their geographical distribution (north, central and south).

In some cases, low values of allocated funds were in agreement with the percentages of hazardous areas and buildings at risk, such as for Veneto and Friuli Venezia Giulia. These regions were mainly involved with other natural events, such as floods and earthquakes. The trend was opposite for the Valle d’Aosta and Liguria regions (Fig. 7a in blue). Both of them had a high portion of their territory subject to landslide hazard, but few earmarked funds. Instead, Lombardy and Piedmont (which are partially plain areas) showed high values of funds, with intermediate values of percentage of landslide hazard areas and buildings at risk. This may be linked to the urbanisation and the spread of the population on the floodplain in the southern part of these regions. Conversely, landslides were concentrated in the northern part of the region along the Alpine arc, where they caused many fatalities.

In central Italy, a more homogeneous distribution of funds can be recognized, with values ranging from 15 to 54 million €. The percentage of hazardous area ranged from 0.16% in Umbria to 1.2% in Tuscany, while the percentage of buildings at risk showed 0.05% and 0.51% (Fig. 7b in green) in the same regions. Emilia Romagna and Abruzzo followed the Tuscany region, presenting high values of landslide hazard (approximately 1% for the first and approximately 0.55% for the second). In these regions the high percentage of hazard entailed a high percentage of buildings at risk, 0.43% and 0.28%, respectively. The high percentages in the Emilia Romagna region agreed with the high values of allocated funds (32.6 million €, Fig. 7 in green).

The Southern Italy was the portion of Italy with more earmarked funds (Fig. 7 in light blue). The earmarked funds agreed with the high percentage of landslide hazards in the Campania region. In general, the percentage of landslide hazard areas varied from a minimum of almost 0.11% in Calabria to a maximum of 0.88% in Campania. Campania, Molise and Basilicata were the regions with the highest percentage of landslide hazard areas in the southern region. Furthermore, the allocated funds were in concordance with the percentage of buildings at risk in all southern regions. The Calabria, Sicily and Campania regions exhibited the highest percentages of buildings at risk, 0.29%, 0.39% and 0.70%, respectively.

This analysis revealed that the southern regions had more funds for soil defence in the observed period. In contrast, the number of news related to landslides, the percentages of territory subject to landslide hazards and of buildings at risk are sensibly lower than those of other parts of Italy.

### Warning hydrological zones (WHZs)

**Figure 8:**
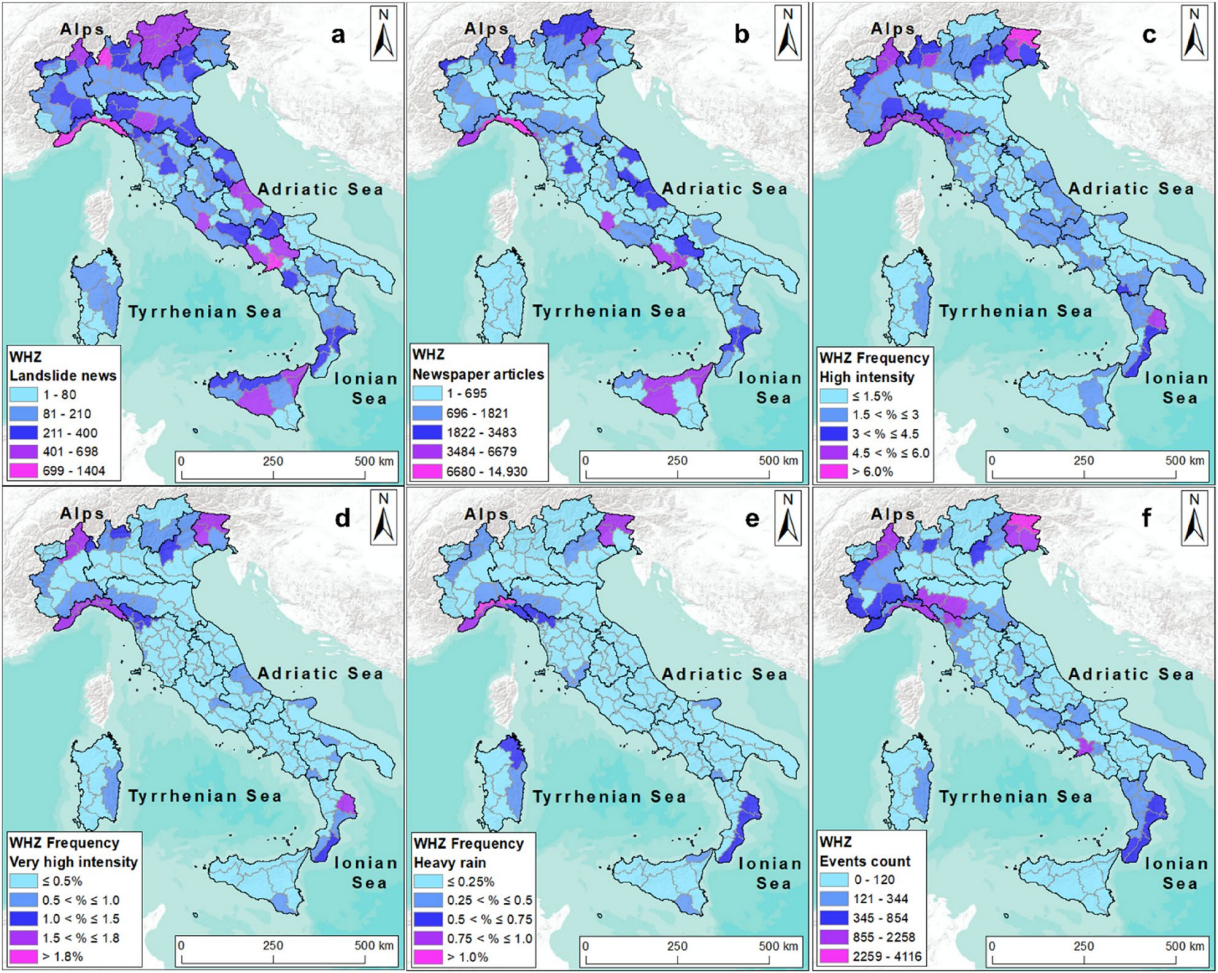
Italy divided into 158 Warning hydrological zones (WHZs). In (**a**) the distribution of “landslide news”; (**b**) the distribution of media impact with “newspaper articles”; (**c**–**e**) rain frequencies “high”, “very high” and “heavy rain”; and (**f**) event sums of the three classes or event counts. The map was generated using ESRI ArcMap 10.8.1 (https://www.arcgis.com/home/item.html?id=33064a20de0c48d2bb61efa8faca93a8).

For civil protection purposes, the knowledge of the rain distribution is fundamental when preparing national weather bulletins. A combination of rain distribution data with “landslide news” and “newspaper articles”, at the WHZ scale, can provide helpful outcomes for those involved in landslide management and prevention. “Landslide news” and “newspaper articles” were mainly located in the northern part of Italy and along the Apennines (Fig. 8a,b). In general, the number of “landslide news” presented a direct correlation with the number of “newspaper article” for each WHZ. An exception can be recognized for one WHZ in the northern part of the Puglia region, where a low number of “landslide news” was associated with a relatively higher number of ”newspaper articles”, indicating that landslide events had a high media echo in this area.

The areas less involved by landslides were located along the northeastern coast and in Puglia. A similar distribution was recognized by Del Soldato et al.^50^ when considering rainfall frequency. Indeed, the northern Italy showed a very good correspondence between the variable “newspaper articles” and rainfall data, with emphasis in the Liguria region, northwestern portion of the Alps (Valle d’Aosta and Piedmont) and the northeast (Trentino Alto Adige, Veneto and in part of Friuli Venezia Giulia).

Central Italy was the region where the main differences were recognized between the different classes of relevant rainfall (Fig. 8c–e). In fact, the classes “heavy rain” and “very high intensity” were more concentrated in the Liguria region and in the northern part of Tuscany, while “high intensity” class was more evenly distributed along the central regions.

A good correspondence was highlighted between “newspaper articles” and the intensity classes as well as with “event count” (Fig. 8f) in the northern WHZs of Tuscany and Emilia Romagna and in the central Italy with Marche, Abruzzo and Lazio.

In the southern Italy, the highest values of “landslide news”, “newspaper articles” and rainfall data were located along the Ionian seacoast, in the WHZs of the Calabria region and in one WHZ of the southeastern part of the Basilicata region. The Puglia region did not show high values of “landslide news”. However, one WHZ with a higher media impact (Fig. 8b) was revealed, even if associated with low frequencies of relevant rainfall events.

**Figure 9:**
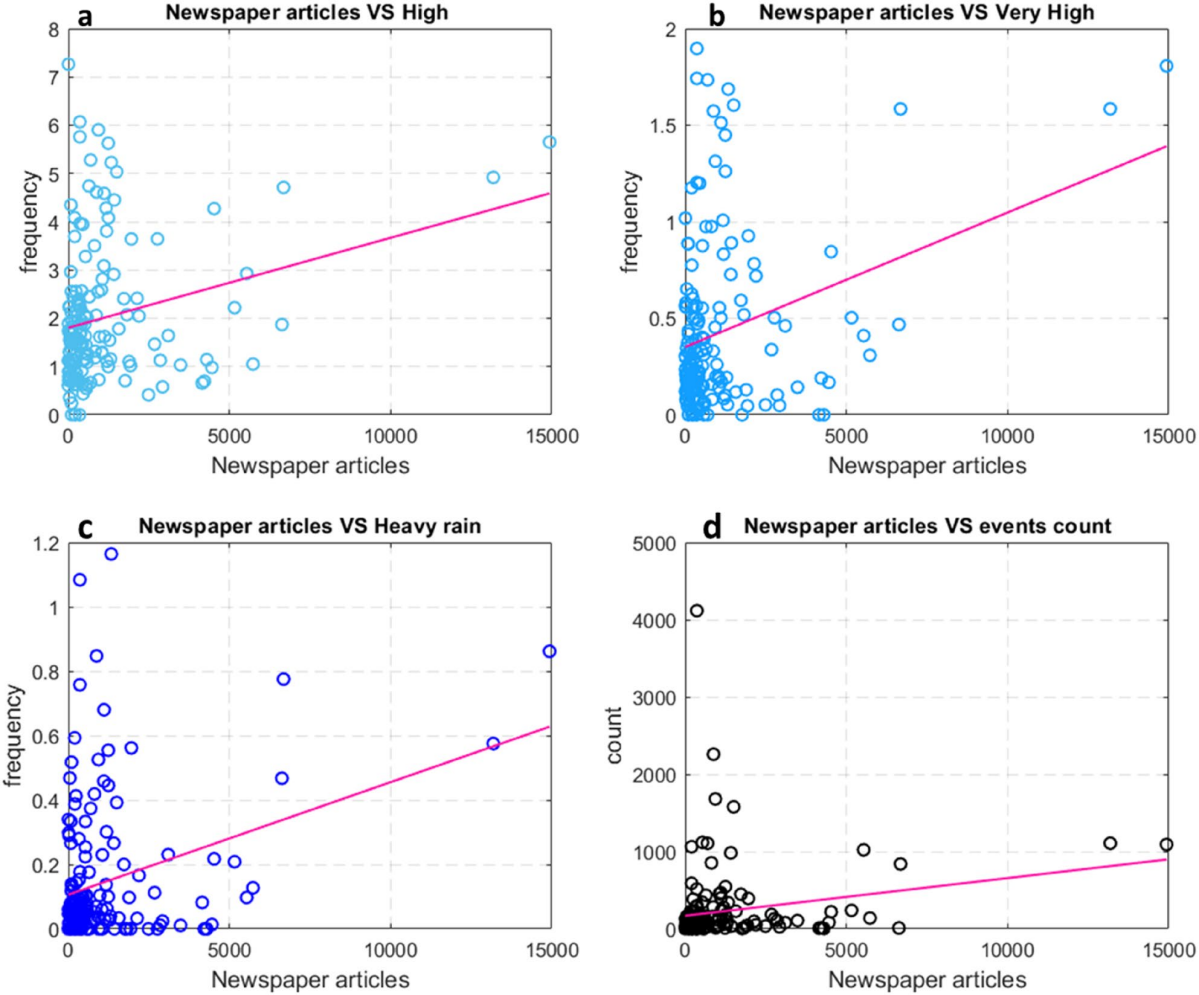
Distribution of “newspaper articles” with different frequencies of rainfall intensity for each WHZ. In (**a**) “newspaper articles” and “high” intensity; (**b**) “newspaper articles” and “very high” intensity; (**c**) “newspaper articles” and “heavy rain”; (**d**) the correlation between “newspaper articles” and the events count.

A more detailed analysis was conducted to obtain the correlation between the rainfall data and the number of “newspaper articles”. Figure 9 displays the correlation between “newspaper articles” and rainfall intensities (Fig. 9a–c) and the event counts (Fig. 9d) for each WHZ. Although low values of media impact (as “newspaper articles”) sometimes corresponded to low values of rainfall intensity and vice versa, it was not possible to outline a clear correlation.

Since the data do not follow a Gaussian distribution, two nonparametric correlation indices were used to verify the rate of correlation between the analysed parameters. The Kendall’s and Spearman rank correlation coefficients resulted in low values, as reported in Table 1 confirming the presence of a slight correlation between the parameters.

**Table 1:** Results of the nonparametric test for the analysed variables.

	Kendall	Spearman
Newspaper articles—high intensity rainfall	0.15	0.22
Newspaper articles—very high intensity rainfall	0.09	0.14
Newspaper articles—heavy rain	0.13	0.19
Newspaper articles—event counts	0.20	0.29

## Discussion

In this work, different data sources were used to obtain information about landslide events and direct consequences in Italy for 10 years (2010–2019). A landslide inventory derived from social media was used as a base proxy to correlate rainfall data and impacts of landslides in an attempt to show how social media in combination with other sources can be utilized to assist government authorities with a better knowledge of the landslide hazard of a territory.

Four datasets with different information were explored: (i) online newspapers, (ii) rainfall data, (iii) population at risk for landslides and floods in Italy (Polaris database) and (iv) earmarked funds for remediation work by the National Repository of Soil Defence interventions (ReNDiS database). At the same time, the total number of published articles for each event was considered to outline the media impact or intensity of the landslide events.

The identification of controlling factors of landslide distribution and occurrence is difficult because the relationship between landslides and the causative components varies spatially and temporally^52^. Nevertheless, a full understanding of these factors is relevant for the assessment of natural hazards^53^ and of their direct effects in terms of human lives and earmarked funds for soil protection. For this reason, rainfall data were analysed and correlated at different scales to “landslide news” and “newspaper articles”.

From a temporal point of view, each Italian region experienced some landslides in the investigated period, with approximately 1477 IDEMs per year. 2014 had 2988 “landslide news”, and a continuous increase until 2019 was observed. This peak of news in 2014 was attributable, among other things, to the distribution over a long time interval of news about the Mont de La Saxe landslide in the Valle d’Aosta region. The Mont de La Saxe landslide was a rock-fall type landslide, and it had many reactivations, causing damage or leading to road closures. Consequently, each time it reactivated, new articles were published. During 2014, rain gauges measured 1007 mm/year of rainfall, and 19 regions were involved in landslides, causing almost 3406 IDEMs. According to the Polaris report^54^, 2014 had several landslide phenomena that affected large areas. On January 19–20, two different weather perturbations involved several zones in Liguria and Emilia Romagna causing death, injuries and damage into the railway network. On May 3rd, an area of Marche was affected by intense rainfall, triggering many landslides and causing damage mainly to the road network. The same scenario occurred on August 2nd in some provinces of the Veneto region. From September 3 to 6, in the northern part of Puglia, approximately 600 mm of rainfall was recorded, triggering several debris flows and mudflows. This amount of rain was very significant considering that the mean annual rainfall of these areas is approximately 800 mm/year. From 9 to 15 October, many provinces in Liguria, Tuscany, Emilia Romagna, Piedmont and Friuli Venezia Giulia were affected by the same perturbation. Many landslides were triggered, causing damage and human losses. On November 10–15, a similar meteorologic event occurred in northern Italy, involving provinces in Liguria, Lombardy and Piedmont and leading to more damage. Considering the ReNDiS database, it was possible to derive the year of intervention financing. It is reasonable to argue that funds for the events were distributed in years after the landslide. For example, the increase of earmarked funds from 2015 to 2017 can be referred to previous events (e.g., those happened in 2014, as in Campania, Emilia Romagna, Lazio, Liguria, Lombardy, Marche, Piedmont and Tuscany).

The highest number of “landslide news” was recorded in 2019, associated with a mean annual rainfall of 904 mm/year, a value 12% higher than the average rainfall of the climatic reference period, 1961–2019^55^. In the same year, several events with long temporal distributions and involving large areas were also reported by the Polaris report^56^. Between 11 and 12 June 2019 in the Lombardy region an extreme rainfall event (characterized by 125.6 mm in 12 h) led to the triggering of many landslides. From 19 to 22 October Lombardia, Liguria and Piemonte were involved in a very heavy intensity storm causing many landslides, including debris flows. Consequently, damage to structures and infrastructure as well as victims, casualties and dozens of evacuees were reported. In summary, 2019 can be referred to as the second year with the highest values of IDEMs and rainfall events, 2775 and 4529, respectively.

Generally, the temporal distribution of “landslide news” showed two increases, from 2010 to 2014 and then from 2015 until 2019. A similar trend was confirmed by Franceschini et al.^40^, who showed that the average number of days with landslides increased from 3 to 5 after 2014. Rainfall data followed a different distribution; in fact, rain data recorded a decrease from the first quinquennium to the second one. Conversely, the distribution and number of victims remained constant over the 10 years. These results are partially in accordance with the outcomes of Crozier^2^, UN^3^ and Porfiriev^4^. These authors highlighted an increasing trend in the number of natural disasters and high-intensity rainfall events, with a consequent increase in the proportion of natural hazards, damage, and losses.

The spatial distribution of “landslide news” and “newspaper articles” highlighted that the regions most impacted by landslides were mainly in northern Italy, along the Alps and in western regions. This trend agrees with the rainfall distribution and IDM number. On the other hand, the earmarked funds (from the central government) for soil protection were higher in southern Italy than in northern Italy. This distribution is in accordance with the outcomes of the work of the World Bank, United Nations^57^ and Padli et al.^58^. In these studies, the authors pointed out that regions with lower social capital (such as the southern regions of Italy) may also have weak economic structures, thus experiencing difficulties in securing adequate resources to recover the damage from natural disasters. Campania, Sicily, Puglia, Basilicata and Calabria exhibit a significantly lower index of economic well-being than the northern regions^59^. The same regions, revealed high values of percentages of infrastructure at landslide risk^47^. This may have resulted, as a consequence, to a sharp increase in prevention activities for soil protection in recent years. For example, Campania exhibited the highest number of buildings at risk, consistent with a high value of hazardous area. As expected, it was the area with the most funds allocated for soil protection. Another example, the Basilicata region, revealed low percentages of hazard areas, in contrast with its geological characteristics. In fact, it consists of land that is easily subject to erosion and runoff. Consequently, the loss of vegetation and land cover has also led to serious instability phenomena. To face this issue, the region has opted for a policy of prevention and rehabilitation and an afforestation and hydraulic-forestry rehabilitation programme^60^. In both examples, the distribution of earmarked funds for soil protection is consistent with the goals of prevention and the recovery of damages caused by landslide events.

It must be pointed out that the distribution of funds for soil protection depends not only on the hazard level of an area and on the number and intensity of landslides but also on local or national political scenarios and on the available social capital. Political processes are quite complex and not straightforward; hence, the distribution of funds could be made considering not only the environmental parameters. This complexity could explain the poor correlation between the earmarked funds and the hazard level of the Italian regions.

The central regions of Italy presented high values of “landslide news”, “newspaper articles” and a high frequency of relevant rainfall events. This aspect was related to the Apennine chain, which crosses the country from north to south and is formed mainly of arenaceous flysch^29,61,62^ and is historically affected by landslides.

In general, Liguria, Lombardy, Campania, Sicily, Tuscany and Emilia Romagna were the regions with the highest number of “landslide news”. Puglia with 202 and Basilicata with 402 are the regions with the fewest publications. This trend agreed with the elevated values of hazardous areas as a in function of regional size, except for Liguria and Sicily.

The divergent distribution of some variables in the Friuli Venezia Giulia, Trentino Alto Adige, Umbria, Puglia, Basilicata, and Calabria regions was linked to the occurrence of localized and very intense or sometimes extreme precipitation. Extreme weather events can trigger landslides in uninhabited areas causing low media impacts and IDMs. Otherwise, as in the case of Umbria, Lombardy and Trentino Alto Adige, one single event or a few events can result in high IDMs. Some authors^63–66^ have assumed that the increased occurrence of extreme events, even localized events, is caused by climate change. Loayza et al.^67^ recently stressed that natural disasters cause significant economic and physical losses, whose effects could spread beyond the immediate locality.

For civil protection purposes, combining different data sources at detailed scale can enhance the awareness of disaster managers. The 158 WHZs divide Italy on the basis of morphology, catchment boundaries and administrative limits. An analysis was applied to obtain more details about the spatial distribution news and of relevant rainfall events. A good correlation can be recognized between “newspaper articles” and event counts, but not with the frequency of relevant rainfall events. The absence of correlation can be due to intrinsic characteristics in the publication news. The publication of one news item and its consequent media impact in different newspapers depends on the landslide event. Some areas can be involved with high intensity precipitation events but cause landslide phenomena with a low involvement of urbanized areas and therefore few published articles. On the other hand, a single rainfall event, even in an area with a low frequency of relevant rainfall events, can trigger a landslide with high human involvement. Consequently, many articles can be published, creating a significant media impact.

This work gave the opportunity to make an overview of landslide hazards in Italy, but some limitations need to be recognized. The main limitations are linked with the use of online newspapers as the source for landslide data. Inside newspaper articles some technical details, such as the type of landslide and its dimension or volume, are often missing. From this kind of source, the exact location of the landslide is a parameter rarely available^40^. Moreover, there is a claim that mass media attention is not uniformly distributed across disaster-affected areas^68^. The presence/absence of news is affected by some factors, such as disruption in communication services, sociodemo-graphic factors (the events affecting socially vulnerable populations receive less attention), the lack of exposed elements (roads, habited areas, etc., reporting low media impact) involved in landslides or the continued reactivation of one landslide over time (e.g., La Saxe landslide; for each reactivation, more articles were published^40^). All these factors can bias the real distribution of landslide events and their use for local scale analyses can lead to the overestimation or underestimation of the real hazard of specific zones.

For these reasons, the use of newspaper articles may be useful for analyses over large areas but not to create a detailed landslide inventory or for detailed analyses^40^.

## Conclusion

Italy has the highest cumulative number of deaths or missing people because of landslides and the highest expected yearly loss of life in Europe^16^. Few studies have been produced on the combination of social media and other information available to assess landslide hazards over a region. In this work, four datasets were used to obtain temporal and spatial information about landslide events in Italy. Datasets of newspaper articles were used as a proxy for landslide hazard estimation. This dataset was correlated with rainfall data, affected people and reported expenses for soil protection measures. Identifying the factors controlling landslide distribution and occurrence is difficult because the relationship between landslides and the causative factors varies spatially and temporally. Nevertheless, a full understanding of these factors is vitally important for the assessment of natural hazards and their effects in terms of fatalities and earmarked funds for soil protection.

“Landslide news” showed an increasing trend from 2010 to 2014, which was repeated in the 2015–2019 period, in contrast with rainfall data, “newspaper articles” and reported expenses, while the IDEMs number remained constant. The spatial distribution revealed that there were more “landslide news”, “newspaper articles”, and IDEMs in the northern regions and in some cases in southern regions (Campania and Calabria). A similar trend was found in the frequency of relevant rainfall events. Conversely, the distribution of earmarked funds was more concentrated in southern Italy than in northern Italy.

The increase in prevention activities for soil protection in recent years, in southern and partially in central Italy, can be linked to high percentages of landslide hazards and buildings at risk that characterise these areas.

Despite the known and demonstrated limitations of social media data with respect to validated official reports, this study confirms that relevant and statistically significant information on landslide hazard can be obtained by data-mining of social networks during emergencies. Such data, properly filtered and classified, may be of notable help in increasing our present capability of calibrating and validating early warning models, with particular reference to data-scarce areas and back-analysis of undocumented past events. Furthermore, these evaluations can represent a useful tool to understand and assess the impact of natural disasters, as well as to plan the best strategies for risk reduction at regional or national scale.

## Data Availability

The data from social media about landslide events and Rainfall data are available from the corresponding author upon reasonable request. Polaris data were obtained from several online reports (https://polaris.irpi.cnr.it/report/). ReNDiS data for landslide events were obtained from an online database (http://www.rendis.isprambiente.it/rendisweb/vistepub.jsp).
